# Essential role of the *BRCA2B* gene in somatic homologous recombination in *Arabidopsis thaliana*

**DOI:** 10.5114/bta.2023.132773

**Published:** 2023-12-21

**Authors:** Padinhatta Purayil Amritha, Jasmine M. Shah

**Affiliations:** Department of Plant Science, Central University of Kerala, Kasaragod, Kerala, India

**Keywords:** *Arabidopsis thaliana*, *BRCA2B* gene, detector line, somatic homologous recombination

## Abstract

Constant exposure to various environmental and endogenous stresses can cause structural DNA damage, resulting in genome instability. Higher eukaryotic cells deploy conserved DNA repair systems, which include various DNA repair pathways, to maintain genome stability. Homologous recombination (HR), one of these repair pathways, involves multiple proteins. BRCA2, one of the proteins in the HR pathway, is of substantial research interest in humans because it is an oncogene. However, the study of this gene is limited due to the lack of availability of homozygous *BRCA2*-knockout mutants in mammals, which results in embryonic lethality. *Arabidopsis thaliana* has two copies of the *BRCA2* homologs: *BRCA2A* and *BRCA2B* . Therefore, the single mutants remain nonlethal and fertile in *Arabidopsis*. The *BRCA2A* homolog, which plays a significant role in the HR pathway of germline cells and during the defense response, is well-studied in *Arabidopsis*. Our study focuses on the functional characterization of the *BRCA2B* homolog in the somatic cells of *Arabidopsis*, using the homozygous *ΔBRCA2B* mutant line. The phenotypic differences of *ΔBRCA2B* mutants were characterized and compared with wild *Arabidopsis* plants. The role of *BRCA2B* in spontaneous somatic HR (SHR) was studied using the *ΔBRCA2B*-gus detector line. *ΔBRCA2B* plants have a 6.3-fold lower SHR frequency than the control detector plants. Expression of four other HR pathway genes, including *BRE*, *BRCC36A*, *RAD50*, and *RAD54*, was significantly reduced in *ΔBRCA2B* mutants. Thus, our findings convey that the *BRCA2B* homolog plays an important role in maintaining spontaneous SHR rates and has a direct or indirect regulatory effect on the expression of other HR-related genes.

## Introduction

DNA repair is a biological process by which cells routinely identify and correct genetic damage. This regular maintenance of genome integrity is crucial for the survival of all organisms. Environmental factors and metabolic activities can cause deleterious lesions, like doublestrand breaks, which adversely affect genome stability and must be repaired rapidly (Tiwari and Wilson, 2019; Alhmoud et al., 2020). Various DNA repair pathways, such as homologous recombination (HR), nonhomologous end joining (NHEJ), mismatch repair (MMS), base excision repair (BER), and nucleotide excision repair (NER), are known to correct DNA damage (Chatterjee and Walker, 2017; Alhmoud et al., 2020). The activation of the repair pathway depends on the nature of the DNA damage. For example, double-strand breaks (DSBs) are repaired by two DNA repair pathways, NHEJ and HR. While NHEJ is a rapid, error-prone pathway that does not require long homologous sequences, HR is a slow, homology-based, error-free DNA repair mechanism (Shen and Li, 2022; Lam, 2022).

The phase of the cell cycle, the presence of sister chromatids, and the mode of resection determine which DSB repair pathway must be chosen, whether HR or NHEJ (Frigerio et al., 2023). In mitotic cells, the HR pathway is activated during the S and G2 phases, during which the sister chromatids are available to provide the region of homology. At the DSBs, the broken DNA is resected to form 3’ single-stranded (ss) DNA overhangs, which are recognized by either the HR-specific protein Replication Protein A (RPA) or the CTC1-STN1-TEN1 (CST) complex. Additionally, RAD51-mediated strand exchange is exhibited in the CST-coated ssDNA at high ionic strength (Lei et al., 2021). In contrast, NHEJ is activated during the G0 and G1 phases of the cell cycle, where the sister chromatids are not available and DSB resection activity is downregulated (Lamarche et al., 2010).

HR is a well-characterized DNA repair pathway in mammals (Krejci et al., 2012). The HR pathway involves several steps: detection of DSB, activation of protein kinases, activation of cell cycle checkpoints, recruitment of proteins involved in the HR pathway to the break site, and, finally, DSB repair (Lamarche et al., 2010). HR serves dual functions in two different types of cells: it facilitates DNA repair in somatic cells and develops genetic variability during meiosis in germline cells. In plants, many genes related to the HR pathway have been studied and characterized using various T-DNA insertion mutants. The HR pathway genes such as *RAD54*, *RAD51*, *ATM*, *XRCC*, and *BRCA2A* have been well-characterized in *Arabidopsis thaliana* (Garcia et al., 2000; Osakabe et al., 2006; Durrant et al., 2007; Wang et al., 2010; Da Ines et al., 2013).

*BRCA2* is a prominent member of the HR pathway (De Picciotto et al., 2016). In mammals, *BRCA2* is highly expressed in the S-phase, and improper functions of the *BRCA2* gene can result in replication errors in somatic cells that may later transform into cancer (Roy et al., 2012). In humans, *HsBRCA2* is a potent oncogene (Roy et al., 2012) and is involved in releasing RAD51 protein into the ssDNA (exposed during DSB or at the site of stalled replications), coated with RPA protein (Holloman, 2011). Given that mutations in *HsBRCA2* result in a high risk of cancer, this gene has been well-characterized in humans (Venkitaraman, 2019). In *Arabidopsis*, the *BRCA2* gene exists as two homologs: *AtBRCA2A* and *AtBRCA2B* (Pfeffer et al., 2017). A previous study on double homozygous insertion mutants of *AtBRCA2A* showed a 30% reduction in spontaneous somatic homologous recombination (SHR) frequency compared to wild plants (Wang et al., 2010). Both *AtBRCA2* genes are required for meiotic as well as somatic HRs (Siaud et al., 2004; Seeliger et al., 2012). Both sister loci seem to play a redundant role in somatic recombination (Seeliger et al., 2012). In this study, we report the influence of the *BRCA2B* gene in maintaining spontaneous SHR rates and regulating the expression of other HR pathway genes in *A. thaliana* plants.

## Materials and methods

### Plant materials

The *A. thaliana* Columbia (Col-0) ecotype and the *BRCA2B* mutant (SALK_124404) seeds were purchased from the *Arabidopsis* Biological Resource Center and provided by R. Baskar (Indian Institute of Technology – Madras, India). The mutation detector line R2L1, in Col-0 background (Li et al., 2004), was obtained from Francois Belzile (University of Laval, Canada). The detector *BRCA2B-gus* mutant was generated by crossing homozygous plants from the R2L1 and SALK_124404 lines. This was followed by the selfing of the F1 progeny to obtain homozygotes for both loci.

### Arabidopsis growth conditions

*A. thaliana* seeds were surface-sterilized, germinated, and grown as previously described (Joseph et al., 2019). Two-week-old plants were carefully transferred to standard vermicompost, purchased from the Central Plantation Crop Research Institute, Kasaragod, India.

### Confirmation of the T-DNA insertion mutant of AtBRCA2B gene

**Fig. 1:**
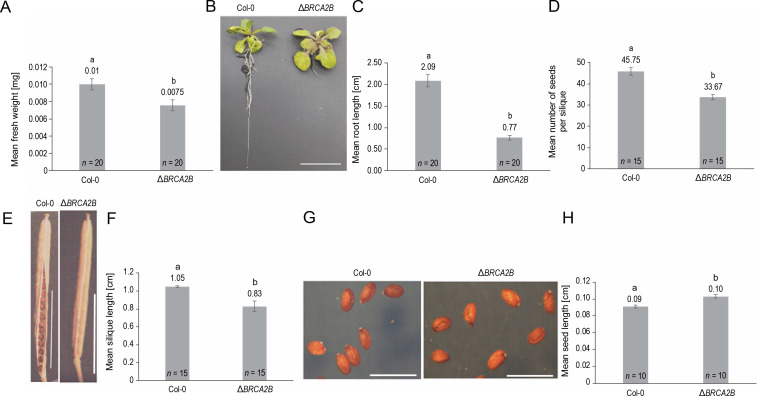
Comparison of morphological characteristics of wild type (Col-0) and the *Arabidopsis* mutant *ΔBRCA2B* (A) fresh weight, (B) 2-week-old plants displaying the root lengths (scale bar – 1 cm), (C) mean root lengths of 2-week-old plants, (D) a mean number of seeds, (E) a silique of the wild type and mutant plant (scale bar – 0.5 cm), (F) mean length of the siliques, (G) mature seeds of the wild type and mutant plants (scale bar – 0.01 cm), (H) mean length of the seeds; error bars represent the ± standard error; ^a,b^ indicate that the mean values are significantly different at *P* < 0.01 as determined by the Student’s *t*-test; *n* – number of plants observed in each experiment

The *BRCA2B* mutant line, SALK_124404, is a homozygous mutant in which the T-DNA was inserted into the 13^th^ intron (supplementary Fig. 1A). The mutant was reconfirmed by PCR (supplementary Fig. 1C) using T-DNA-specific primers (supplementary Fig. 1A and Fig. 1B) (O’Malley et al., 2015) and by sequencing the amplicon using the Sanger sequencing method, executed by Xcelris, India (Crossley et al., 2020) (supplementary Fig. 1D).

### Making of the GUS detector, BRCA2B-gus mutant line

The *GUS* detector, *BRCA2B-gus* mutant line, was generated by crossing the plants from line R2L1 with *BRCA2B* mutant plants. The *GUS* gene in R2L1 is a noncoding gene into which two identical introns are inserted in reverse orientation (supplementary Fig. 2A). Recombination events are monitored by *GUS* expression due to the HR that occurs between the introns. The double homozygous *BRCA2B-gus* mutant line has two T-DNAs (supplementary Fig. 2B). To identify such events, sequential steps of screening and confirmation were followed in each generation (supplementary Fig. 3A). Crossing R2L1 with *BRCA2B* mutants developed heterozygous F1 progenies. Selfing of F1 progenies resulted in F2 progenies. All F2 plants were germinated on kanamycin-containing growth media. Kanamycin-resistant F2 plants were screened for the homozygosity of *BRCA2B* loci using PCR with BP/RP (supplementary Fig. 4A) and LP/RP (supplementary Fig. 4B) primer pairs. Identified F2 plants homozygous for the mutant *BRCA2B* loci were screened using PCR with *GUS* primers to check whether they have the second T-DNA harboring the *GUS* recombinant construct (supplementary Fig. 4C). To identify the F2 plant homozygous for *gus* loci, we performed semi-quantitative real-time PCR of the DNA with NPTII* primers (supplementary Fig. 4D). Homozygotes for both T-DNAs would have four copies of *NPTII*, and those that are hemizygous for the *gus* loci would have three copies of *NPTII* (supplementary Fig. 3B). The identified double homozygous mutant was re-confirmed by segregation analysis of its F3 generation using PCR with *GUS* and T-DNA-specific primers (supplementary Fig. 5A–5C).

### Phenotype analysis

Phenotype analysis was conducted in three experimental replicates, each with 15–20 plants. The root length was measured manually. Fresh weight was assessed using a weighing balance (Shimadzu, Japan). The lengths of seeds, siliques, and rosettes were measured digitally using a stereo microscope (Motic, Spain). The number of seeds per silique was counted manually. Chlorophyll extraction was performed using DMSO, and the optical density was measured at 645 and 663 nm in a UV spectrophotometer (Thermo Scientific, USA) against a DMSO blank (Hiscox and Israelstam, 1979). Chlorophyll content was calculated based on Arnon’s equation (Pérez-Patricio et al., 2018).

### Extraction and quantification of RNA/DNA, and cDNA synthesis

RNA was isolated from 4-week-old plants using Tri reagent (Origin, Diagnostics & Research, India) (Shi and Bressan, 2006). DNA was extracted using the CTAB method (Doyle and Doyle, 1987) from 4-week-old plants. Both nucleic acids were quantified using the Nanodrop 2000c Spectrophotometer (Thermo Scientific, USA). cDNA was synthesized using the Moloney murine leukemia virus reverse transcriptase (M-MLV-RT) kit (Invitrogen, USA) from 1 μg of the isolated RNA samples, following the manufacturer’s protocol.

### Primer designing

The T-DNA-specific primers used to screen the *AtBRCA2B* loci (supplementary Fig. 1B) were as recommended by the Salk Institute Genome Analysis Laboratory. The remaining primers (supplementary Table 1) were designed using the PrimerQuest tool from IDT (https://www.idtdna.com/pages/tools/primerquest). All primers were synthesized by Sigma-Aldrich India Pvt Ltd.

### PCR screening and sequence confirmation

The PCR reaction mixture contained 100 ng of genomic DNA, 1 μl (10 pmol/μl) each of forward and reverse primers, 2.5 μl of Taq polymerase (1U)-containing master mix, and nuclease-free water. All PCR components were from Origin Diagnostics & Research, India, and the experiments were run on an Eppendorf Mastercycler-PCR Thermal Cycler. PCR conditions were as follows: initial denaturation for 2 min at 94°C, denaturation for 1 min at 94°C, annealing (temperature based on the primer used), extension for 1 min at 72°C, and cooling for 5 min at 4°C. Confirmation was achieved by sequencing the amplicon using the Sanger sequencing method from Xcelris, India (Crossley et al., 2020).

### Somatic recombination assay

Recombination assays were conducted using homozygous plants from the GUS detector mutant and the *BRCA2B* mutation detector lines. Somatic mutations were detected in 4-week-old plants by the occurrence of blue spots, obtained after GUS histochemical staining as previously described (Jefferson, 1989; Shah et al., 2015). The experiments were repeated three times, with more than 100 plants in each trial. Images of plants with blue sectors were captured using a stereo microscope (Motic, Spain).

### Real-time PCR

For expression analysis, *UBC9* was used as the reference gene, and PCR conditions were described previously (Joseph et al., 2019). Semiquantitative real-time PCR (Haurogné et al., 2007) was used for the zygosity confirmation of the double homozygous *BRCA2B-gus* detector mutant. For this, 100 ng of total DNA was subjected to real-time PCR using *NPTII* * primers (supplementary Fig. 4D).

All reactions were performed using the Essential DNA Green Master (Roche Diagnostics India Pvt Ltd) master mix, and the experiments were run on the Roche LightCycler^®^ 480 II system. The reaction mixture for expression analysis comprised 1 μl cDNA/DNA, 10 μl master mix, 1 μl (10 pmol/μl) of each forward and reverse primer, and nuclease-free water.

### Statistical analysis

The phenotypic characters, spontaneous SHR rates, and real-time expression of repair genes were statistically analyzed using the Student’s *t*-test (Kalpić et al., 2011) with Microsoft Excel 2016. The significance of the data was determined at *P* < 0.01 for phenotypic characters and *P* < 0.05 for spontaneous SHR and real-time expression.

## Results

### BRCA2B mutation results in altered morphology of Arabidopsis

Mutation in the *AtBRCA2B* gene induced distinct changes in plant morphology compared to wild plants (Fig. 1). The mutant plants exhibited significantly reduced fresh weight (1.3-fold) (Fig. 1A), root length (2.7-fold) (Fig. 1B, Fig. 1C), average number of seeds per silique (1.4-fold) (Fig. 1D), and silique length (1.3-fold) (Fig. 1E, Fig. 1F) in comparison to wild plants. In contrast, seed length in mutants was 1.1-fold longer than in control plants (Fig. 1G, Fig. 1H). Neither chlorophyll content nor rosette diameter showed any significant variation (supplementary Fig. 6A, Fig. 6B).

### Confirmation of the GUS detector, BRCA2B-gus mutant line

To understand the influence of the *AtBRCA2B* gene on spontaneous SHR in plants, we developed a double homozygous mutant line ( *BRCA2B*-*gus*) by crossing the *BRCA2B* mutant line SALK_124404 with the mutation detector *Arabidopsis* line R2L1 (Li et al., 2004). The R2L1 line serves as an *in planta* intrachromosomal recombination assy system, where the SHR rate can be monitored using the mutated *GUS* gene, visualized as blue sectors on the plant after histochemical staining with 5-bromo-4-chloro-3-indolyl glucuronide (X-gluc) (Li et al., 2004). Twenty-five kanamycin-resistant F2 plants were screened for homozygosity of *BRCA2B* loci using PCR with T-DNA screening primer pairs. Three F2 plants (numbers 5, 15, and 18) were identified as homozygous for the mutant *BRCA2B* loci because they yielded the expected amplicon with BP/RP primers and did not amplify with the LP/RP primers (supplementary Fig. 1A, Fig. 4A, Fig. 4B). Plants that amplified with both primer pairs were hemizygous for this locus. Plants 5, 15, and 18 also produced the expected amplicons when screened using *GUS* primers, indicating that they harbor the second T-DNA containing the *GUS* recombinant construct (supplementary Fig. 4C). F2 plants homozygous for *gus* loci were screened using semiquantitative real-time PCR of DNA with *NPTII* * primers. Homozygosity was confirmed by the variation in CP value proportional to the number of *NPTII* gene copies. Of the three F2 plants, plant number 15 had a lower CP value (supplementary Fig. 4D), indicating higher copies of the *NPTII* gene. Loci segregation was re-confirmed in the F3 generation; all F3 plants displayed results expected of a homozygote double mutant (supplementary Fig. 5A–5C).

### BRCA2B mutants display a hypo-recombination phenotype

**Fig. 2:**
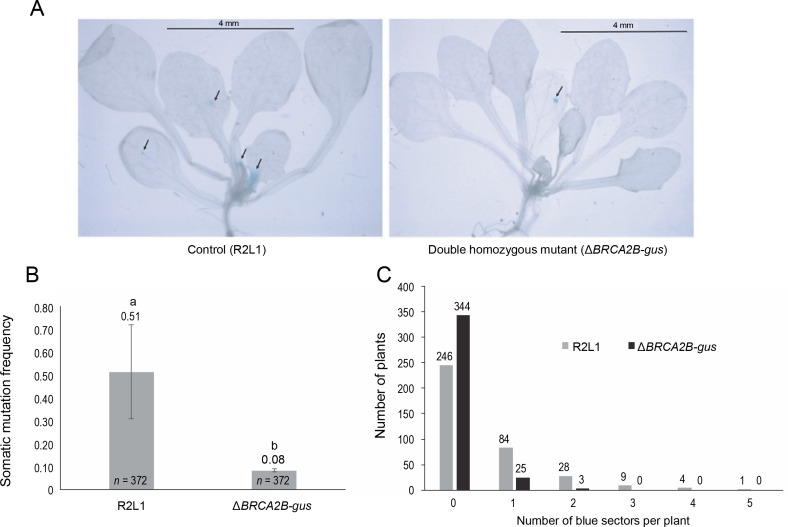
Somatic homologous recombination (SHR) analysis (A) Image of control (R2L1) and *ΔBRCA2B-gus* double homozygous mutant plant after GUS-histochemical staining (scale bar-4 mm); black arrows indicate blue sectors developed on the plants (B) graphical representation of somatic mutation frequency in control (detector line) and double homozygous mutant plant; error bars represent the ± standard error and ^a,b^ represent statistically significant differences in SHR rate at *P* < 0.05 as determined by Student’s *t*-test; *n* – total number of plants observed (C) graphical representation of the number of blue sectors in individual plant

Similar to the R2L1 lines, the double homozygous mutant *BRCA2B*-*gus* also exhibited blue sectors upon GUS histochemical staining (Fig. 2A). A significant reduction in the SHR frequency (6.3 fold) was observed in the *BRCA2B*-*gus* mutants compared to the control R2L1 plants (Fig. 2B). Furthermore, the analysis of blue sector distribution within individual plants revealed a higher number of blue sectors per plant in the control R2L1 plants (Fig. 2C). A maximum of only two blue sectors per plant were observed in *BRCA2B*-*gus* mutants, compared to five in the R2L1 control plants. Approximately 92% of the *BRCA2B*-*gus* mutants did not exhibit any spontaneous SHR in the *GUS* recombination region, in contrast to 66% of the control R2L1 plants. Consequently, our results suggest that *BRCA2B* is essential for inducing spontaneous SHRs in plants.

### BRCA2B mutation downregulates the expression of other genes in the HR pathway

**Fig. 3:**
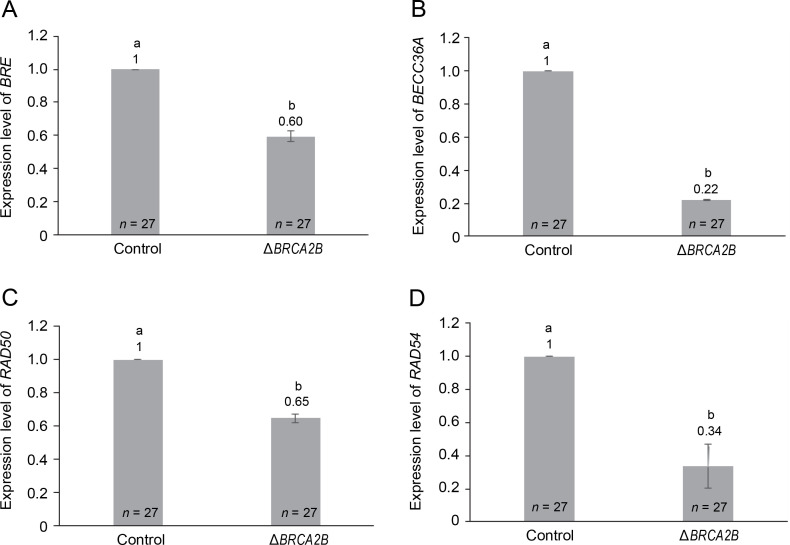
Comparison of relative expression levels of genes involved in homologous recombination pathway, in the wild type and *BRCA2B* mutant *Arabidopsis* plants (A–D) represent comparative expression levels of genes *BRE*, *BRCC36A*, *RAD50,* and *RAD54*, respectively; error bars represent ± standard error; symbols a and b indicate that the expression values are significantly different as determined by the Student’s *t*-test at *P* < 0.05; the data represent the mean of the three independent trials; *n* – total number of plants

To further examine the influence of *BRCA2B* on the HR pathway, we explored the expression of four additional genes – *BRE, BRCC36A, RAD50*, and *RAD54* – within the HR pathway (Fig. 3). Among these, *BRE, BRCC36A*, and *RAD50* operate upstream of *BRCA2B* in the HR repair pathway, while *RAD54* functions downstream (D’Amours and Jackson, 2002; Mazin et al., 2010; Biswas et al., 2018). All four HR genes, namely *BRE*, *BRCC36A*, *RAD50*, and *RAD54*, demonstrated a significantly diminished expression, with respective fold changes of 0.60, 0.22, 0.65, and 0.34, in the *BRCA2B* mutants (Fig. 3A–3D). This suggests that the presence or absence of *BRCA2B* is required for regulating other HR proteins. Further, the overall reduction in the SHR rates in *BRCA2B*-*gus* mutant plants might be attributable not solely to the absence of the *BRCA2B* protein but also to the decreased availability of *BRE*, *BRCC36A*, *RAD50*, and *RAD54* proteins.

## Discussion

*BRCA2* plays a pivotal role in the HR pathway, prominently functioning in double-strand break repair (Roy et al., 2012). *HsBRCA2*, recognized as a tumor suppressor gene and widely regarded as an oncogene, has mutations associated with the onset of breast and ovarian cancers in humans (Paul and Paul, 2014). Germline mutations in the human *HsBRCA2* gene are correlated with a heightened risk of developing breast cancer (Pietschmann et al., 2005). In mice, mutations in *BRCA2* lead to early embryonic lethality, and surviving mice manifest sterility in adulthood (Jensen et al., 2013). Although the DNA repair pathway is highly conserved across higher eukaryotes, mutations in *BRCA2* genes are nonlethal in plants (Seeliger et al., 2012). The model plant *A. thaliana* carries two homologs of the *BRCA2* gene, *BRCA2A* (1151 aa) and *BRCA2B* (1155 aa), which share a 94.5% identity. The *HsBRCA2* gene encodes a substantially larger protein comprising 3418 amino acids and shares a 21% identity with the *AtBRCA2A/B* proteins. In *Arabidopsis*, *AtBRCA2A* and *AtBRCA2B* are situated on chromosomes IV and V, respectively, and diverge only in their second BRC repeat (Trapp et al., 2011). In this study, we report the influence of the *AtBRCA2B* gene on *Arabidopsis* phenotype, spontaneous SHR frequency, and other genes within the HR pathway.

We examined the *BRCA2B* mutant line SALK_124404, which exhibited a notable decrease in plant fresh weight, root length, silique length, and seed quantity. However, the seeds of *BRCA2B* mutants were longer than those in wild-type plants. Abe et al. (2009) and Wang et al. (2010) previously reported that other *BRCA2B* mutant lines, namely *atbrca2b*-1 (a Ds mutant, Nossen background), and SALK_037617 (T-DNA insertion mutant, Columbia background), were fertile but did not provide additional phenotypic details. Both studies indicated that the *atbrca2a/b* double mutants displayed severe growth abnormalities, such as hypersensitivity to genotoxic stresses, altered cell cycle progression, and sterility under various DNA damaging stresses. Previous reports also revealed that the *atbrca2a/b* double mutants (Dumont et al., 2011) and RNAi-silenced mutants for the *AtBRCA2A/B* genes (Siaud et al., 2004) were sterile and exhibited malformed male and female gametophyte development (Seeliger et al., 2012).

While *AtBRCA2A* and *AtBRCA2B* participate in the HR pathway and are hypothesized to fulfill redundant roles, we aimed to investigate whether *AtBRCA2B* alone contributed to maintaining spontaneous SHR rates. To this end, we created a double homozygous mutant, combining the *BRCA2B* line SALK_124404 with the previously documented *GUS* mutation detector line R2L1 (Li et al., 2004; Shah et al., 2015). SHR was markedly reduced – by 6.3-fold – in these detector mutants. Wang et al. (2010) have previously reported SHR reductions of about 2.5 and 1.1 in *BRCA2A* and *BRCA2B* lines, respectively. Nonetheless, the rates due to *BRCA2B* mutants (1.1-fold) were not significantly lower compared to ours (6.3-fold). This discrepancy may arise because Wang et al. (2010) utilized a different *GUS* mutant line, and the chromosomal position effect on the *GUS* recombination substrate might influence its sensitivity (Ülker et al., 2012). Nonetheless, akin to *AtBRCA2A* (Wang et al., 2010), our results indicate that *AtBRCA2B* is requisite for maintaining spontaneous SHR rates. Comparable reporter lines have been used to screen somatic mutation rates in prior studies by mating with *Arabidopsis* mutants for other DNA repair genes like *AtRAD54* (Osakabe et al., 2006), *AtRAD51D*, *AtRAD51B*, and *AtXRCC* (Da Ines et al., 2013), which also exhibited reduced SHR compared to control plants.

*BRCA2*, a pivotal gene in the HR pathway, has been extensively studied in animal models to deeply probe its functional dynamics. It plays a role in loading the RAD51 protein onto the ssDNA (Davies et al., 2001). RAD51 and DMC1, the two recombinases of the HR pathway, operate in somatic and meiotic cells, respectively. *BRCA2* acts as a positive regulator for both of these recombinases (Kumar et al., 2019). In humans, *BRCA2* interacts with RAD51 via its BRC repeat and also exerts an antagonistic function over FIGL1 (a protein with antirecombinase activity), which subsequently governs RAD51 and DMC1 (Kumar et al., 2019). A mutation in *BRCA2* is known to activate alternative pathways, such as NHEJ (alt-NHEJ) and single-strand annealing (SSA), and it also possesses an antagonistic effect on Pol and RAD52-mediated RPA displacement from ssDNA, thereby constraining the alt-NHEJ and SSA repair (Han et al., 2017). In a prior study, Wang et al. (2010) reported that *AtBRCA2A* also significantly regulated the transcription of defense-related genes.

Given the regulatory function of *AtBRCA2A* and our findings on SHR rates, we sought to explore whether the absence of *AtBRCA2B* impacted the expression of other HR genes. The expression of four genes – *BRE*, *BRCC36A*, *RAD50*, and *RAD54* – was reduced (by 50% or more) in the *Atbrca2b* mutants. Prior research demonstrated that BRE aids in the localization of the BRCA1 complex to the DSB site during SHR (Feng et al., 2009). Interestingly, in mouse embryonic cells, while *BRCA2* mutations prove lethal, *BRE* gene over-expression can sustain these cells’ viability by facilitating the deubiquitylation of CDC25A phosphatase (Biswas et al., 2018). BRCC36 is a component of the BRCC holoenzyme complex, comprising proteins BRCA1, BRCA2, RAD51, and BRCC36. Working in tandem with BRE, BRCA1, and BARD1, BRCC36 facilitates E3 ligase activity and acts as a regulator of BRCA1 activation during ionizing radiation (IR) exposure, thus downregulation of *BRCC36* expression compromises DNA repair (Chen et al., 2006). *A. thaliana* contains two *BRCC36* homologs: *AtBRCC36A* and *AtBRCC36B* (Block-Schmidt et al., 2011). *RAD54*, known as a chromatin remodeling factor (Heyer et al., 2006), belongs to the SWI2/SNF2 family of helicases, regulating RAD51 (Solinger et al., 2002). Moreover, RAD54 stabilizes nucleofilament formation and strand invasion by RAD51, promotes nucleosome remodeling, and enhances homology search during HR. In *DMC1* mutated plants, meiotic recombination necessitates *RAD54* for *RAD51*-mediated DSB repair (Sanchez-Rebato et al., 2021). RAD50, part of the MREll complex, influences the predisposition to cancer due to its complex interactions with various oncogenes such as *ATM*, *BRCA1*, and *CHK2* (Heikkinen et al., 2003). The expression of *RAD50* and *RAD51* is elevated in tissues comprising actively dividing cells (Gallego et al., 2001; Doutriaux et al., 1998). Thus, all four genes we selected are requisite for maintaining genome integrity, and our study illuminates the influence of the *BRCA2B* mutation on their expression.

In conclusion, our examination of the phenotype, spontaneous SHR rates, and expression analysis of HR genes using mutant *BRCA2B* plants reveals that *AtBRCA2B* is an essential homolog of *BRCA2A*, crucial for maintaining spontaneous HR in *Arabidopsis* plants. Considering that a homozygous *BRCA2* condition is lethal in the mammalian system and given the high conservation of the DNA repair system in higher eukaryotes, plant system-based experiments have provided deeper insights into the functions of this pivotal gene. It could be possible that, similar to plants, *BRCA2* – which is an oncogene – may also influence the expression of other DNA repair genes in animals. A similar strategy could be employed to study other mammalian homologs, where double knock-outs are lethal in animals.

## Supplementary Material

Essential role of the *BRCA2B* gene in somatic homologous recombination in *Arabidopsis thaliana*
