# Supplementary material for: Essential role of the BRCA2B gene in somatic homologous recombination in Arabidopsis thaliana
Source: BioTechnologia (Pozn). 2023 Dec 21;104(4):371–80. doi: 10.5114/bta.2023.132773 (PMC10777725; doi:10.5114/bta.2023.132773)
Supplement: Essential role of the BRCA2B gene in somatic homologous recombination in Arabidopsis thaliana [file BTA-104-4-51758-s001.pdf]

BioTechnologia vol. 104(4) · 2023
Journal of Biotechnology, Computational Biology and Bionanotechnology SUPPLEMENTARY MATERIALS
https://doi.org/10.5114/bta.2023.132773

CC BY 4.0

Essential role of the *BRCA2B* gene in somatic homologous recombination in *Arabidopsis thaliana*

JASMINE M. SHAH*, PADINHATTA PURAYIL AMRITHA
Department of Plant Science, Central University of Kerala, Kasaragod, Kerala, India

Received: 30 January 2023; revised: 15 June 2023; accepted: 29 August 2023

Abstract

Constant exposure to various environmental and endogenous stresses can cause structural DNA damage, resulting in genome instability. Higher eukaryotic cells deploy conserved DNA repair systems, which include various DNA repair pathways, to maintain genome stability. Homologous recombination (HR), one of these repair pathways, involves multiple proteins. BRCA2, one of the proteins in the HR pathway, is of substantial research interest in humans because it is an oncogene. However, the study of this gene is limited due to the lack of availability of homozygous *BRCA2*-knockout mutants in mammals, which results in embryonic lethality. *Arabidopsis thaliana* has two copies of the *BRCA2* homologs: *BRCA2A* and *BRCA2B*. Therefore, the single mutants remain nonlethal and fertile in *Arabidopsis*. The *BRCA2A* homolog, which plays a significant role in the HR pathway of germline cells and during the defense response, is well-studied in *Arabidopsis*. Our study focuses on the functional characterization of the *BRCA2B* homolog in the somatic cells of *Arabidopsis*, using the homozygous *ΔBRCA2B* mutant line. The phenotypic differences of *ΔBRCA2B* mutants were characterized and compared with wild *Arabidopsis* plants. The role of *BRCA2B* in spontaneous somatic HR (SHR) was studied using the *ΔBRCA2B*-gus detector line. *ΔBRCA2B* plants have a 6.3-fold lower SHR frequency than the control detector plants. Expression of four other HR pathway genes, including *BRE*, *BRCC36A*, *RAD50*, and *RAD54*, was significantly reduced in *ΔBRCA2B* mutants. Thus, our findings convey that the *BRCA2B* homolog plays an important role in maintaining spontaneous SHR rates and has a direct or indirect regulatory effect on the expression of other HR-related genes.

Key words: *Arabidopsis thaliana*, *BRCA2B* gene, detector line, somatic homologous recombination

* Corresponding author: Department of Plant Science, Central University of Kerala, Kasaragod, Kerala, India;
e-mail: jasmine@cukerala.ac.in

A

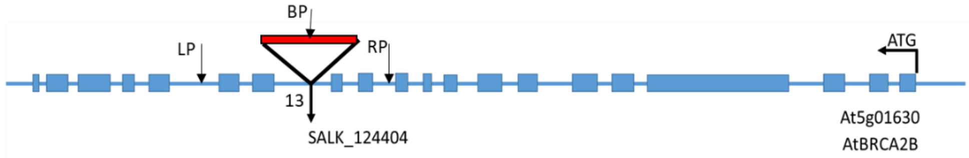

B

Gene name	Accession number	Mutant line	Primer sequence	Annealing Tm (°C)	Amplicon length	
					Wild	Mutant
ATBRCA2B	AT5G01630	SALK - 124404C	LP: 5′ CTTATCTGAAGATCCTCGGGG 3′<br>RP: 5′ CAGCTTAAAGACCTTGGCTCC 3′	60	1003 bp	-
			BP: 5′ ATTTTGCCGATTTCGGAAC 3′<br>RP: 5′ CAGCTTAAAGACCTTGGCTCC 3′	60	-	593bp

C

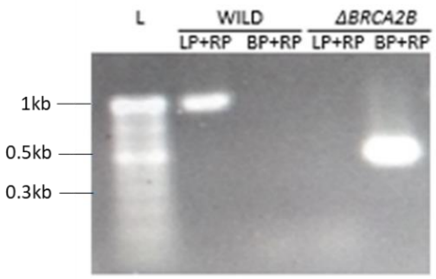

D

GGGGCCTTGGTGGAACACCATTAATTCCTCTCTGGTACTCACACATAATACCCTCAACAAGAGCTGAGCGCCTACGGAAAATAGAAAAAGAAAGAAAGAAAAGTACGAATGAGAATAAACCATGA
GGAACTGCAATCTTTGGCTCTTCTGTTGGCAAAACCATCCATATTCAGAAGGCCAGAAGCTTTTCTAATAAATACAAAGATTCTTTGTCTGTCCCAAAAACTACTTAGAACCCTAATATTGGTGTATGT
AACAAATGTCTCTAGCTTTGATGAGTTAATCTGACACTTCTTAGAAAGTGATGTGCTAAATTGATCACATAAATGTTACTAAAAATTGCTACTCTACAGTTATAAGGAAACTTGCCTCTGATTATGCAGC
TGTATAATTCTACTTTCTATTCTCTCTGATCGAACTATTGACTTCTTTTCACCTAACCTGTATACATCAAGAAAATATAATGCTTTGCGAGAAGTATAAGGAAGACCAAAGTGCATTAACAGGCACACAT
TTATATTGACCTTTCCTTGTATAGGATGGGGTATATTCGTGTGATGCCAGCTAGCGTTTTAGGCACTGGACCCCCATTG

Supplementary Fig. 1. Characterization of the mutant line SALK_124404 (A) Schematic representation of the T-DNA insertion in the *AtBRCA2B* gene with exons (blue boxes) and introns (thin blue lines); the position of T-DNA insertion of SALK_124404 is indicated as a red box in the 13th intron; LP, RP, and BP are the primers used for PCR amplification. (B) Details of T-DNA screening primers and their expected amplicons. (C) PCR confirmation of the homozygosity of the T-DNA insertion mutant. L-1 Kb DNA ladder (D) Nucleotide sequence of PCR product from the mutant. The sequence in red color indicates the T-DNA

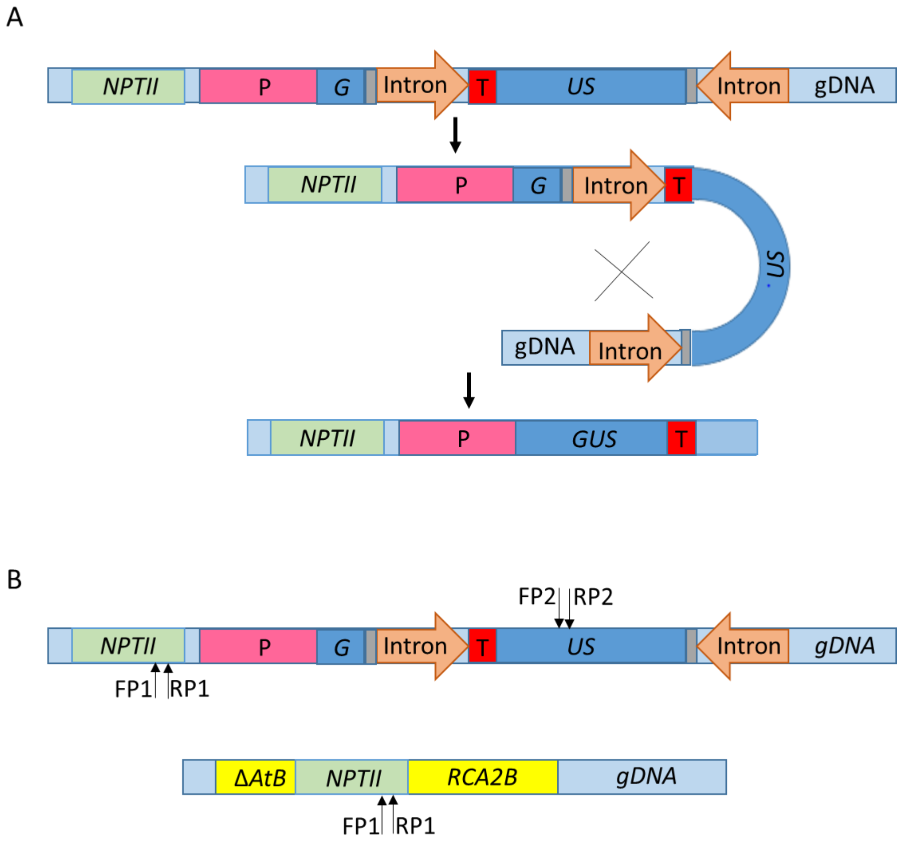

Supplementary Fig. 2. Diagrammatic representation of constructs present in various *Arabidopsis* mutant lines (A) *GUS* recombination construct in the R2L1 detector line, undergoing recombination resulting in the generation of complete *GUS* gene (B) The T-DNAs present in the double mutant Δ*BRCA2B-gus*, showing the locations of various PCR primers also; *NPTII* – Neomycin phosphotransferase encoding gene, FP1-RP1, and FP2-RP2 are primer pairs for *NPTII* real-time PCR and *GUS* PCR respectively; P-promotor, G and US represent truncated *GUS* genes due to the insertion of intron (orange arrow), and T – terminator

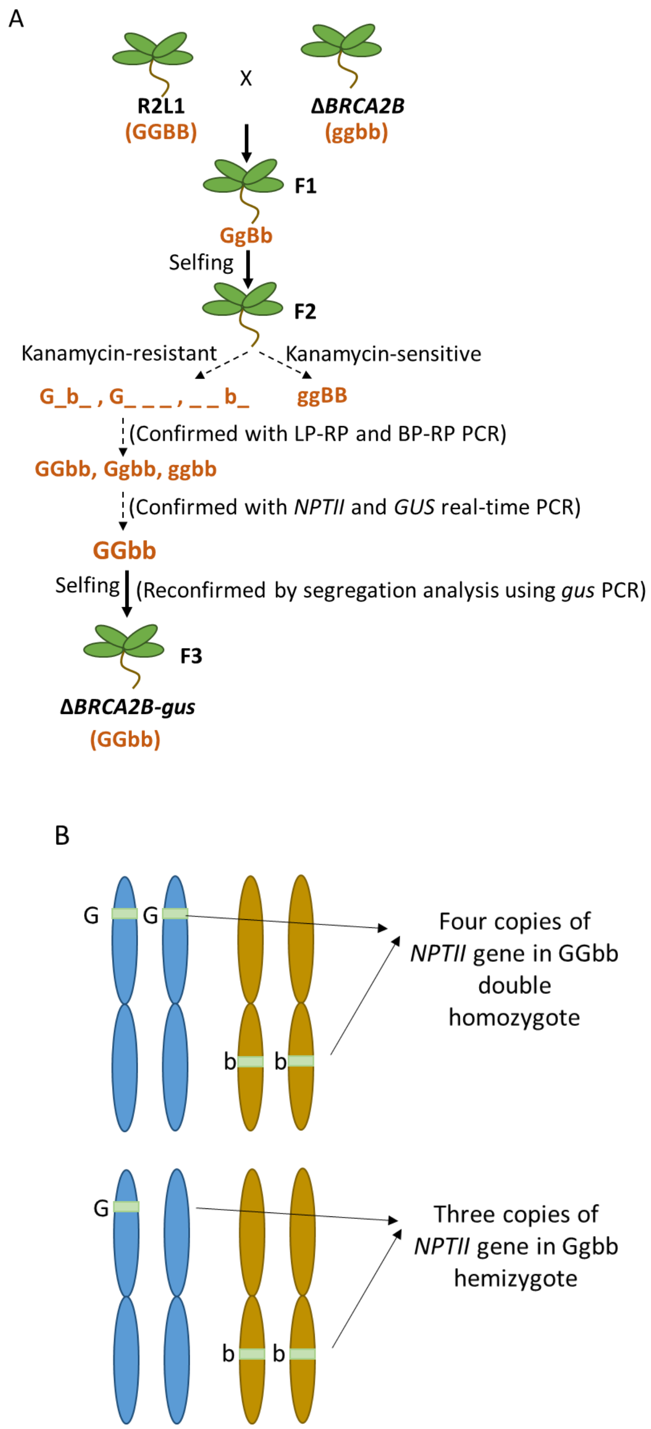

Supplementary Fig. 3. Details of crosses and genotypes of various *Arabidopsis* mutants (A) schematic representation of the crosses, generations, and techniques involved in obtaining the double homozygous mutant Δ*BRCA2B-gus*; the thick black arrows lead to the next generation due to crossing or selfing; the dotted black arrows indicate various steps for screening; (B) diagrammatic representation showing the copy number of the *NPTII* gene, located on two different pairs of homologous chromosomes, in the double-homozygous and hemizygous mutants with the GGbb and Ggbb genotypes, respectively; G and g represent the presence and absence of the *GUS* construct, respectively; B and b represent the normal and T-DNA-inserted alleles of the *BRCA2B* gene, respectively

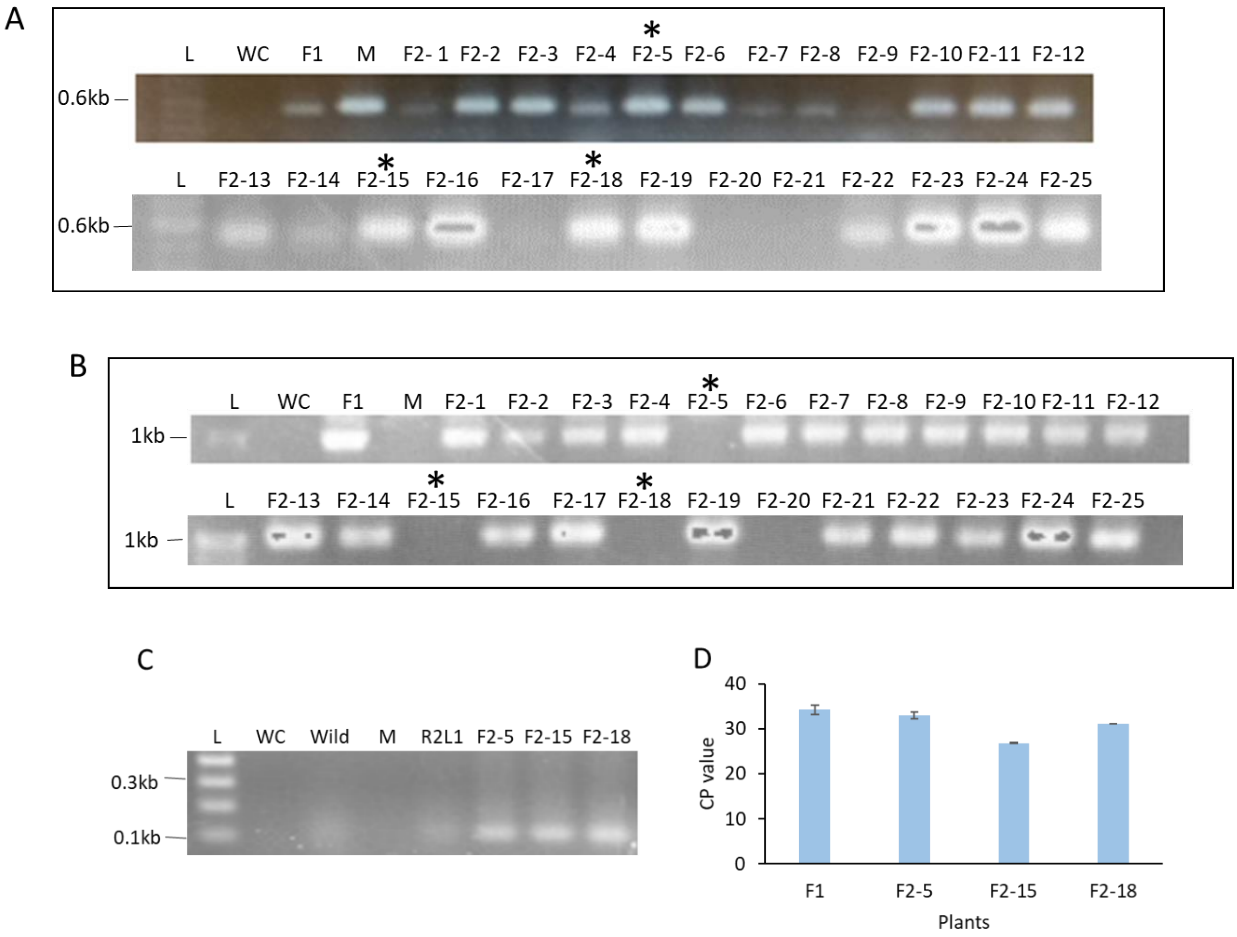

Supplementary Fig. 4. Screening and confirmation of double homozygous mutant *ΔBRCA2B-gus* plants using (A) PCR with BP+RP primers, (B) LP+RP primers, (C) PCR with *GUS* primers; (D) Comparison of CP values of semiquantitative real-time PCR done for screening homozygous *NPTII* loci; L-1Kb DNA ladder, WC – non-template water control, M – homozygous mutant *ΔBRCA2B,* F1-DNA sample of F1 progeny, F2-1 to F2-25-DNA samples of 25 different F2 progeny, the symbol * indicates homozygous loci for T-DNA insertion

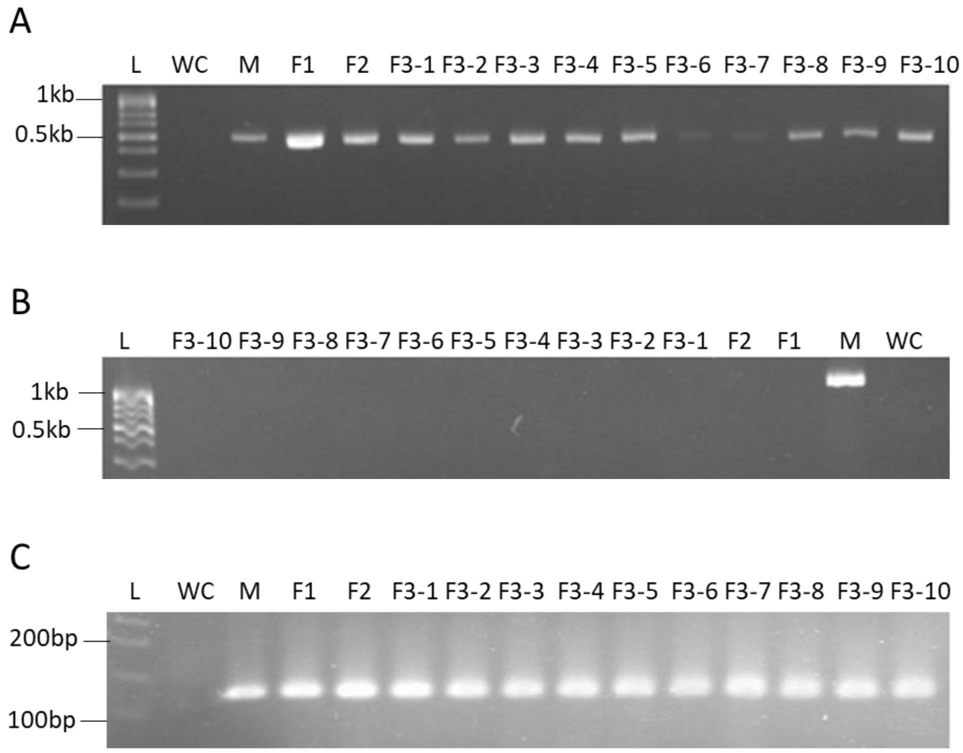

Supplementary Fig. 5. Segregation analysis of plants from F3 generation using PCR with (A) T-DNA-specific primers BP and RP (B) T-DNA-specific primers LP and RP (C) *GUS* primers; F1 and F2 are DNA from the progeny from two generations, F3-1 to F3-10 represent DNA from 10 different F3 progeny; M is the positive control homozygous mutant line *ΔBRCA2B* (A, B), R2L1 (C); L-1Kb DNA ladder (A) and (B), 500 bp ladder (C), WC–non-template water control

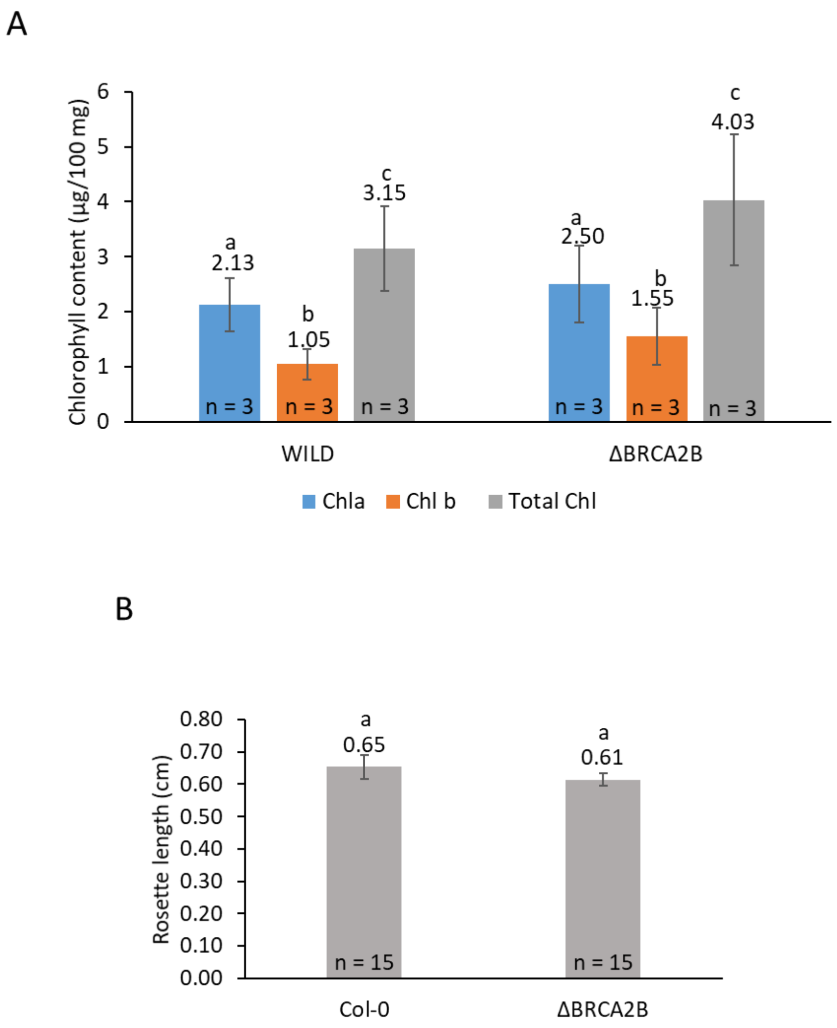

Supplementary Fig. 6. Comparison of morphological characteristics of wild type (Col-0) and the *Arabidopsis* mutant *ΔBRCA2B* (A) chlorophyll content (B) rosette lengths of 2-week-old plants; a, b, and c means denoted by the same symbol indicate that they are not significantly different at $P < 0.01$ as determined by the Student's *t*-test, conducted separately for Chl a – Chlorophyll a, Chl b – Chlorophyll b, and Chl – total chlorophyll; n – number of plants observed

Supplementary Table 1. Primers used in the study, for PCR and real-time PCR

Gene name	Primer sequence (5'–3')	Annealing Tm [°C]	Amplicon length, bp	
			gDNA	cDNA
GUS	F: CGTGGCAAAGGATTCGATAAC<br>R: TCTCTTCAGCGTAAGGGTAATG	56	104	–
*NPTII**	F: CAATAGCAGCCAGTCCCTTC<br>R: TGAATGAACTGCAGGACGAG	64	113	–
*BRE**	F: GTCAGCACCTCGTTTGAAATTA<br>R: CTCAAGTGTTTCTTCAAGATGGG	61	224	136
*BRCC36A**	F: CATGTCTGAAAGTATGCAAGAGG<br>R: GGTGAATTGAGTTGAAGAAGGG	61	210	126
*RAD50**	F: AGAGGTCAAGATATGGATTAC<br>R: CTCTCATTTCAAGTTCTGTATC	54	190	115
*RAD54**	F: GAATCCTGCCAATGATAAAC<br>R: AGAAACCTGTAGACATATACTC	55	180	81
*UBC9**	F: TTCCCTCCAGATTACCCATTTAAG<br>F: TTCAAGATGTCGAGGCAGATG	55	288	122

F – forward primer, R – reverse primer, Tm – temperature, gDNA – genomic DNA, bp – base pair,
*indicate primers for real-time PCR
